# Depression: Biological Non-Pharmacological Interventions. A Review.

**DOI:** 10.1192/j.eurpsy.2024.1118

**Published:** 2024-08-27

**Authors:** F. A. Rodríguez Batista, S. Trufero Miguel, R. Bordón Guerra, A. R. Neyra del Rosario

**Affiliations:** ^1^Department of Psychiatry, Doctor Negrín University Hospital of Gran Canaria; ^2^Department of Psychiatry, Insular University Hospital of Gran Canaria, Las Palmas de Gran Canaria, The Canary Islands, Spain

## Abstract

**Introduction:**

Major depressive disorder stands as one of the most significant mental health issues in the general population. It impacts the patients’ quality of life and increases both morbidity and mortality. Response and tolerability to available pharmacological treatments are often inefficient, sometimes requiring extended periods to achieve acceptable remission through combinations or augmentations. Non-pharmacological approaches constitute an element in the therapeutic options for this mental disorder. In recent years, there has been a growing interest in non-pharmacological biological treatment interventions. Among the principal ones are Electroconvulsive Therapy (ECT), Transcranial Magnetic Stimulation (TMS), Deep Brain Stimulation (DBS), and Vagus Nerve Stimulation (VNS).

**Objectives:**

The aim of this paper is to review the current available literature to expand our knowledge about biological non-pharmacological treatment in depression, particularly ECT, TMS, DBS, and VNS.

**Methods:**

A qualitative review was conducted over the last 5 years, using the Medline database through PubMed. We selected studies in English or Spanish that met the objectives of the review, excluding references in other languages. The scientific evidence obtained was analyzed and synthesized.

**Results:**

There is growing evidence in this area. TMS, whose place in clinical guidelines remains unclear, is a less available treatment but might be considered in patients with moderate to severe depression who cannot receive pharmacological treatment. DBS, which shows good results in treatment-resistant major depressive disorder, achieves response rates greater than 50%. VNS has accumulated studies since its approval for treatment-resistant depression, showing some latency of response but demonstrating improvement persistence for at least two years, although some studies have not clearly shown a benefit. We also found studies demonstrating the effectiveness and favorable cost-benefit balance of ECT.

**Conclusions:**

This review highlights the importance of increasing knowledge in these types of treatments. They have shown significant progress in recent years. We have a better understanding and use of the technique of ECT, while newer options have gained evidence in effectiveness over these years, with improvements facilitating their use in patients with treatment-resistant depression.

**Disclosure of Interest:**

None Declared

